# Different tillage induces regulation in 2-acetyl-1-pyrroline biosynthesis in direct-seeded fragrant rice

**DOI:** 10.1186/s12870-019-1913-9

**Published:** 2019-07-12

**Authors:** Pan Du, Haowen Luo, Jing He, Ting Mao, Bin Du, Lian Hu

**Affiliations:** 1 College of Engineering, South China Agricultural University, Guangzhou, 510642 People’s Republic of China; 2 Key Laboratory of Key Technology for South Agricultural Machine and Equipment Ministry of Education, Guangzhou, 510642 People’s Republic of China; 3 College of Agriculture, South China Agricultural University, Guangzhou, 510642 People’s Republic of China

**Keywords:** Fragrant rice, 2-acetyl-1-pyrroline, Tillage, Proline, Anti-oxidative enzyme

## Abstract

**Background:**

Land preparation is an important component of fragrant rice production. However, the effect of tillage on fragrant rice production is unclear, especially regarding the biosynthesis of 2-acetyl-1-pyrroline (2-AP), which is the main compound of the unique aroma of fragrant rice. This study aimed to explore 2-AP biosynthesis in fragrant rice under different tillage regimes. Three tillage methods were applied in the present study: conventional rotary tillage (CK) as the control, plough tillage (PT), and no-tillage (NT).

**Result:**

Compared with CK, the PT treatment increased 2-AP content in grain, upregulated the activity of ornithine aminotransferase (OAT) and increased contents of 1-pyrroline and pyrroline-5-carboxylic acid (P5C). Furthermore, the PT treatment increased the grain yield and nitrogen accumulation of fragrant rice. Meanwhile, the 2-AP content in the grain produced under the NT treatment was significantly higher than that in the grain produced under both the PT and CK treatments due to the enhancement of proline content and the activities of proline dehydrogenase (PDH) and △1-pyrroline-5-carboxylic acid synthetase (P5CS). However, the present study observed that the overall production of fragrant rice under NT conditions was inferior due to lower yield, nitrogen accumulation, and anti-oxidative enzymatic activities. Moreover, the organic matter content and soil microorganism quantity increased due to PT and NT treatments.

**Conclusions:**

Compared to CK, PT improved fragrant rice grain yield and nitrogen accumulation and induced an increase in OAT activity and led to an increase in 2-AP concentration. No-tillage also produced increased 2-AP content in grain by enhancing PDH and P5CS activities but limited yields and nitrogen accumulation in fragrant rice.

## Background

Rice (*Oryza sativa.* L) is a staple food and a major grain crop for approximately 70% of the population worldwide [1]. As a specialty rice, fragrant rice is famous around the world because of its unique aroma. In recent years, it has been identified that 2-acetyl-1-pyrroline (2-AP) is the key aromatic compound in fragrant rice varieties [2], and it is also clearly established that the majority of 2-AP is of biosynthetic origin [3]. Although the biosynthesis of 2-AP is a complicated process in fragrant rice, many studies about 2-AP biosynthesis have been reported. For example, the study of Yoshihashi [4] demonstrated that proline was the nitrogen source in 2-AP biosynthesis in fragrant rice. The research of Poonlaphdecha [5] revealed that 1-pyrroline was a limiting precursor in 2-AP biosynthesis according to feeding experiments using rice calli cultures. Furthermore, an early study showed that Badh2 decreased the biosynthesis of 2-AP in fragrant rice by encoding betain aldehyde dehydrogenase [6]. More recently, the 2-AP concentration in grain has emerged as one of the characteristics used to evaluate the grain quality of fragrant rice and, therefore, more scientists are studying methods to increase 2-AP in fragrant rice.

**Fig. 1:**
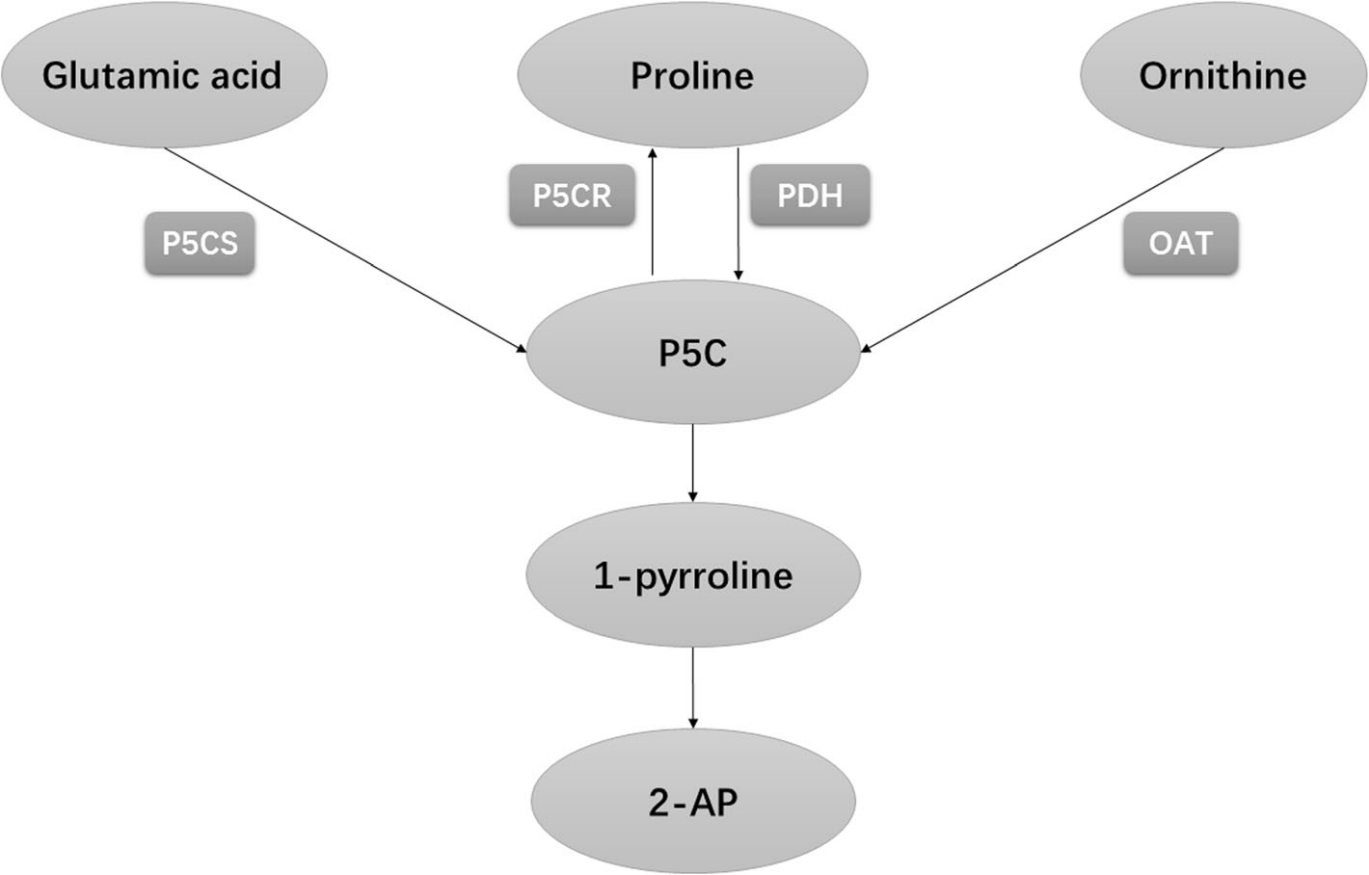
The proposed pathway of 2-AP biosynthesis in fragrant rice (P5CR means Pyrroline-5-carboxylate reductase)

To increase 2-AP content in fragrant rice grain, many crop management strategies have been introduced, such as fertilizer application, water management, and even light control. Previous studies have shown that extra nitrogen applied at the booting stage could induce increases in 2-AP concentrations in fragrant rice [7]. The study of Bao [8] revealed the molecular basis for increased 2-AP content under drying conditions in fragrant rice. Deng [9] demonstrated interacting effects between a mild drought environment and nitrogen dose at the filling stage. Moreover, Mo [10] found that shading during the grain filling stage could greatly increase 2-AP content in fragrant rice. The proposed pathway of 2-AP biosynthesis in fragrant rice was shown in Fig. 1.

Typically, land preparation is the key activity for rice production. However, there has been no research about the effects of tillage on 2-AP biosynthesis in fragrant rice. In agricultural production, soil plays an important role in supporting crop growth and development, maintaining and transporting water and gas, and maintaining nutrient cycling and transformation. Tillage can cut and break the soil layer, make it loose and porous, reduce soil bulk density, improve soil structure, and influence the conditions of soil water, fertilizer, gas, and heat, collectively creating a good growth environment for rice and improving rice yield. There are two tillage methods, plough tillage and rotary tillage, in Chinese agricultural history. In the last decade, most farmers have chosen rotary tillage over plough tillage because of faster land-preparation speed, lower cost, and great soil crushing effect [11]. However, recently, the conventional rotary tillage methods have increasingly attracted farmers and researchers because of soil problems (e.g., poor soil structure) and the negative effect of tillage on soil organisms [12].

Therefore, in order to compare the differential impact of alternative tillage methods on 2-AP biosynthesis in fragrant rice, the present study was conducted in Guangdong Province (a major rice producing province in South China).

## Results

### 2-AP content in grain

**Fig. 2:**
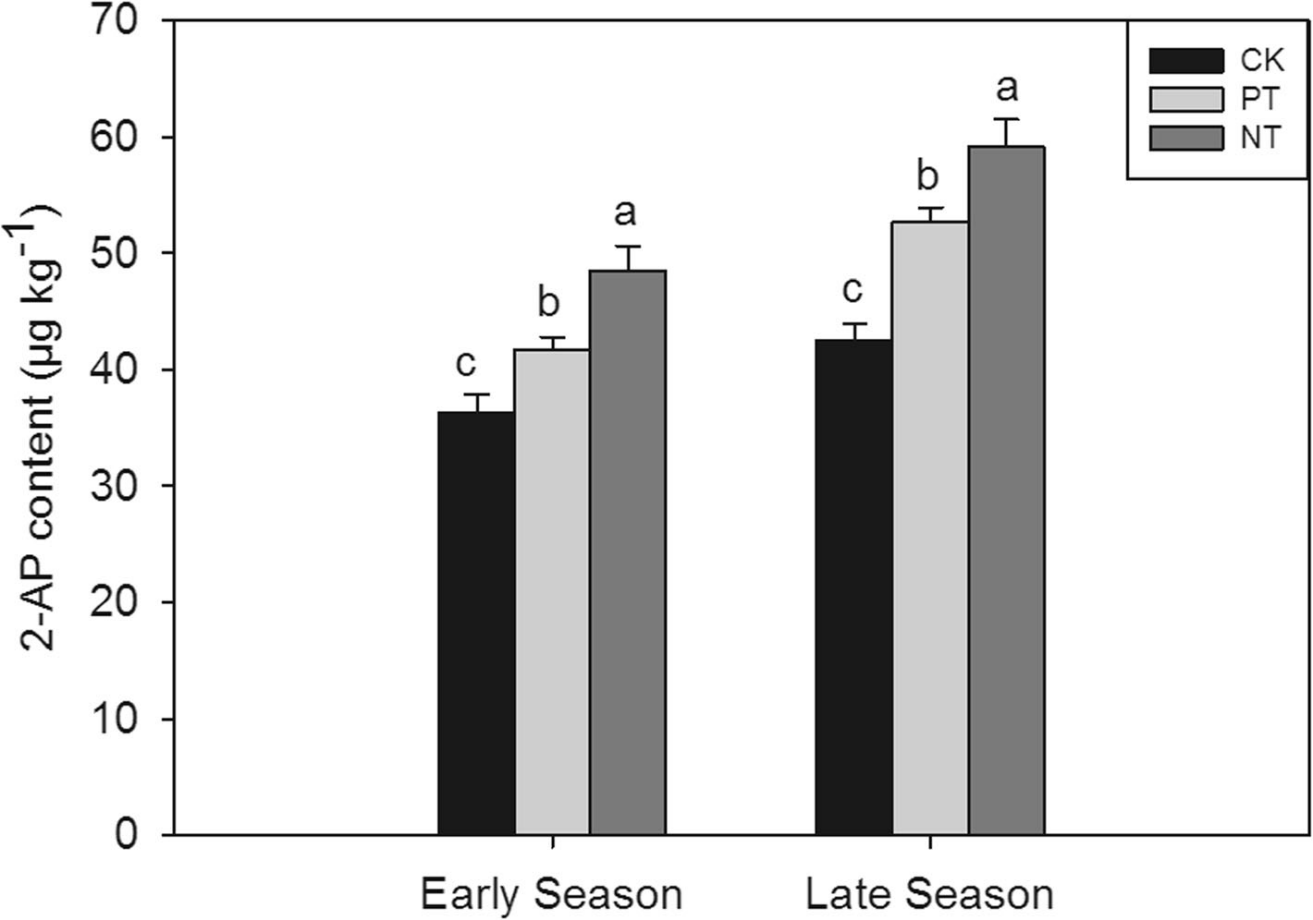
Effect of different tillage on 2-AP content in fragrant rice grain. Capped bars represent S.E. of three replicates. Means sharing a common letter don’t differ significantly at (*P* ≤ 0.05) according to least significant difference (LSD) test. The same as below

Compared to rotary tillage, plough tillage and no-tillage significantly increased 2-AP concentration in grain in direct-seed fragrant rice. As shown in Fig. 2, the 2_AP contents in grain produced with PT and NT were 14.97 and 29.26% higher, respectively, than that observed in grain produced with CK in early the season, while in the late season PT and NT treatments increased 2-AP content in grain by 23.81 and 31.52%, respectively, compared to CK.

### Precursors and enzymes involved in 2-AP biosynthesis

**Fig. 3:**
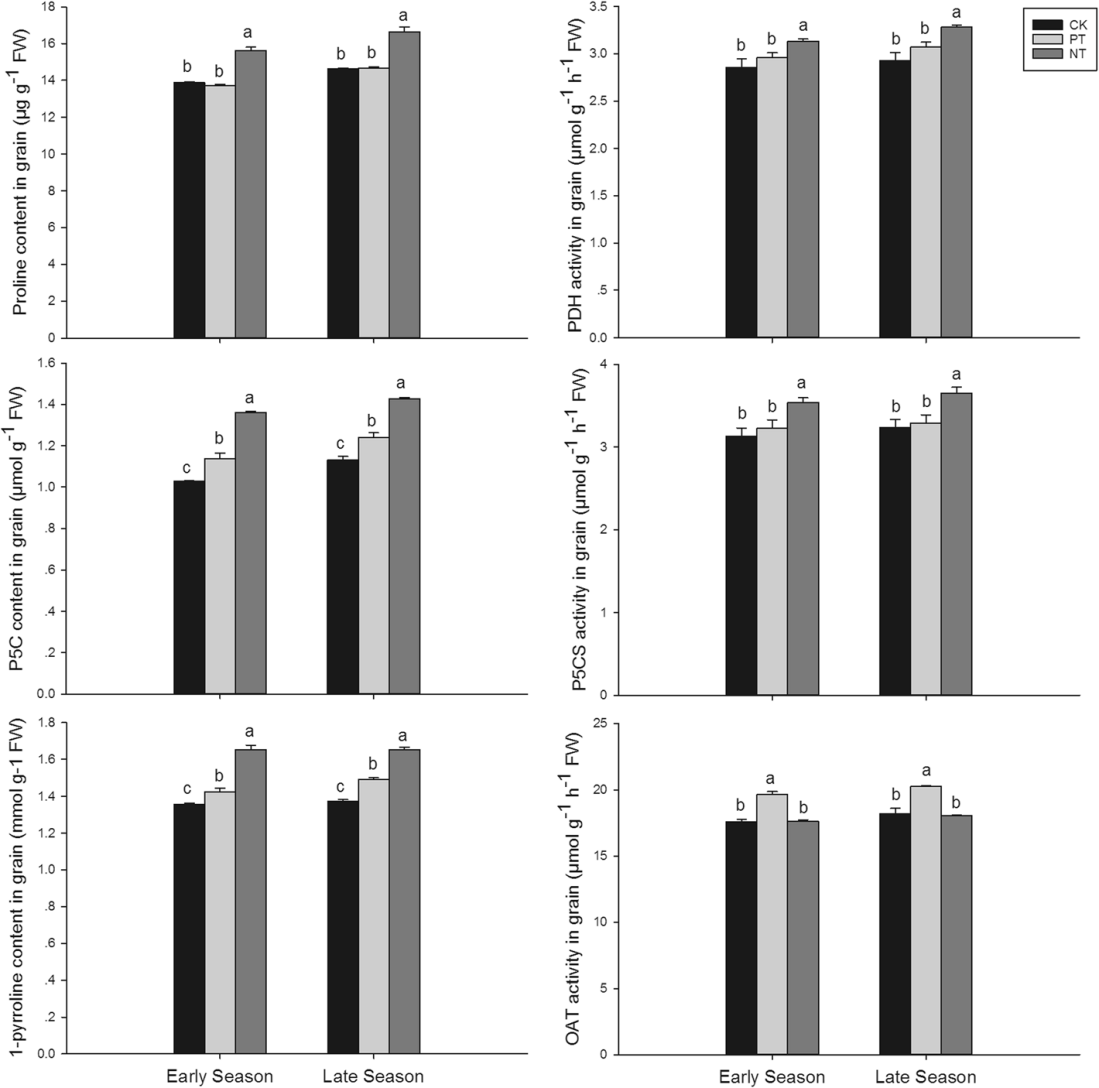
Effects of different tillage types on precursors content and enzymes activities in 2-AP biosynthesis in fragrant rice grain

As shown in Fig. 3, PT and NT treatments affected precursors involved in 2-AP biosynthesis, such as proline, P5C, and 1-pyrroline. Compared to CK, NT significantly increased proline content in grain at the filling stage while there was no remarkable difference between PT and CK in both seasons. The trends of P5C and 1-pyrroline content in grain at the filling stage were both recorded as NT > PT > CK in both seasons. Furthermore, There were some differences among alternative tillage types in terms of enzyme activity involved in 2-AP biosynthesis in fragrant rice (Fig. 3). Compared with CK, NT treatment significantly increased PDH and P5CS activities by 9.45–11.57% and 12.68–12.70%, respectively. There was no significant difference between CK and PT in both the PDH and P5CS activities in both seasons. However, 11.79 and 11.35% higher OAT activities were recorded in PT compared to CK in the early season and late seasons, respectively, while there was no significant difference between NT and CK in OAT activity in both seasons.

### MDA content and anti-oxidant responses in leaves

**Fig. 4:**
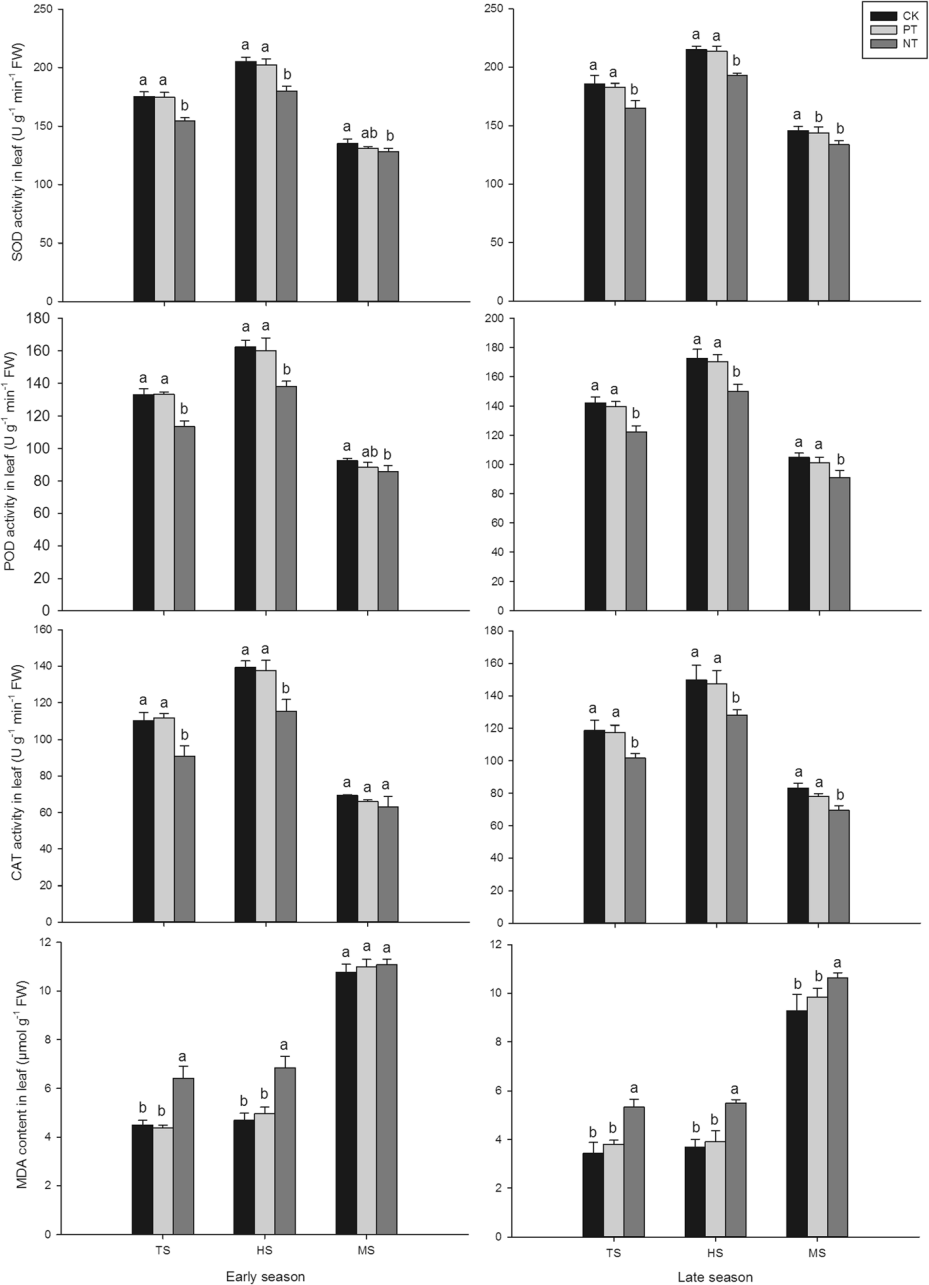
Effects of different tillage on POD, SOD, and CAT activities and MDA content in fragrant rice leaves (TS indicates tillling stage, HS indicates heading stage and MS indicates maturity stage)

As shown in Fig. 4, plough tillage did not greatly affect the anti-oxidative enzymatic activities in fragrant rice, while no-tillage significantly influenced the anti-oxidative enzymatic activities in terms of SOD, POD and CAT compared to rotary tillage. There was no remarkable difference between PT and CK tillage in activities of SOD, POD, and CAT and the content of MDA at the tilling stage, heading stage, and maturity stage for both seasons. However, compared with CK, NT treatment significantly decreased the SOD, POD and CAT activities in both seasons (except CAT activity in maturity stage in the early season). Moreover, higher MDA contents were also recorded in the NT treatment compared to CK in both seasons (except at maturity stage in the early season).

### Soil organic matter and microorganisms

As shown in Table 1, compared with CK, NT and PT treatments increased organic matter content in three soil depth. At 0-10 cm soil depths, the higher number of fungi and actinomycetes were recorded in PT and NT than CK. At 10-20 cm soil depth, there was no significant difference between CK and PT in fungi number while NT and PT treatment remarkably increased bacteria and actinomycetes number. At 20-30 cm soil depth, the trends of bacteria, fungi and actinomycetes number were recorded as: NT > PT > CK.

**Table 1:** Effects of different tillage on soil organic matter content and microorganism community quantity in late season

Soil depth (cm)	Treatment	Organic matter content (g Kg^− 1^)	Bacteria (10^5^ CFU g^− 1^)	Fungi (10^3^ CFU g^− 1^)	Actinomycetes (10^4^ CFU g^− 1^)
0–10	CK	19.654c	2.165b	1.014c	1.802b
	PT	26.729b	2.179b	2.653b	2.064a
	NT	29.583a	2.298a	2.916a	2.087a
10–20	CK	15.599c	1.182b	0.808b	1.028c
	PT	23.246b	1.249a	0.783b	3.098b
	NT	25.942a	1.266a	0.815a	3.266a
20–30	CK	11.858c	0.763c	0.364c	0.872c
	PT	16.727b	1.211b	2.460b	1.615b
	NT	17.311b	1.312a	2.692a	1.718a

Values sharing a common letter within a column don’t differ significantly at (*P* ≤ 0.05) according to least significant difference (LSD) test

### Grain yield and nitrogen accumulation

**Fig. 5:**
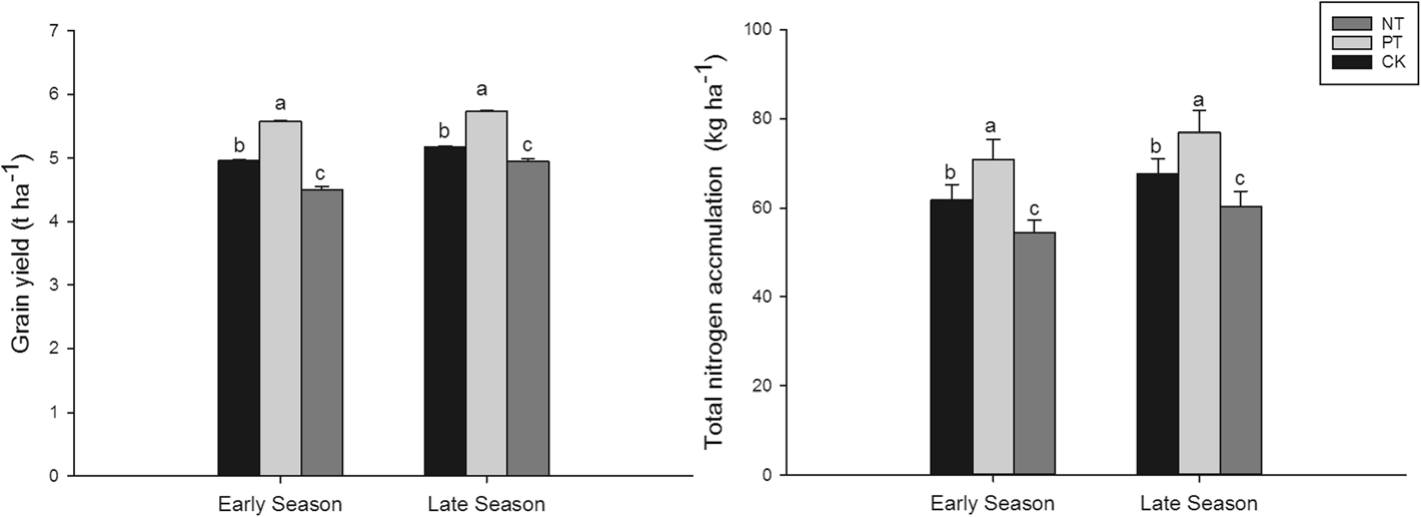
Effects of different tillage types on fragrant rice grain yield

There were some differences among different tillage conditions in fragrant rice grain yield (Fig. 5). Compared to CK, grain yield in the early season and the late season were significantly increased (by 12.30 and 10.62%, respectively) due to PT treatment. However, 8.20 and 3.90% lower grain yields were recorded in NT compared to CK in the early season and the late season, respectively. Furthermore, the trend of total nitrogen accumulation in the early season was recorded as: PT > CK > NT, and a similar trend was also observed in the late season.

## Discussion

The biosynthesis of 2-AP is a very complicated phenomenon in fragrant rice, involving a number of precursors and enzymes, such as proline, 1-pyrroline, P5C, P5CS, PDH, and OAT. In recent years, it is generally accepted that there are three steps to biosynthesize 2-AP in fragrant rice. First, glutamate, proline, and ornithine are converted to P5C by the activities of P5CS, proline dehydrogenase (PDH), and OAT, respectively. Then, P5C reacts with methylglyoxal, which is non-enzymatic, to convert 1-pyrroline, and △^1^- pyrroline is finally produce to 2-AP (Fig. 1) [26, 27].

To increase the 2-AP content in fragrant rice grain, a few studies have investigated the impact of fertilizer application and water management. For example, the investigation of Mo [28] revealed that extra silicon fertilizer could increase the 2-AP concentration in grain by increasing the activity of PDH. Ren [29] demonstrated that there was an interaction between nitrogen application and water management on 2-AP biosynthesis in fragrant rice. The present study documented the improvement of plough tillage and no-tillage on 2-AP content in fragrant rice. For plough tillage, higher P5C, 1-pyrroline content, and OAT activity compared to those for rotary tillage were observed in our study. For no-tillage, the increased 2-AP concentration could be explained by increases in P5CS, PDH activity and proline, P5C and 1-pyrroline content. It seems that plough tillage and no-tillage enhanced 2-AP biosynthesis through different enzymatic pathways.

The difference between plough tillage and no-tillage might be due to the different field environments caused by different tillage [30]. As an important precursor in 2-AP biosynthesis in fragrant rice, the biosynthesis of proline was shown to be greatly affected by the environment [31, 32]. The investigation of Szabados [33] revealed that there are two ways to synthesize proline in plants; these are named the glutamic acid pathway and the ornithine pathway according to the different initial substrates. When nitrogen supply is sufficient, the ornithine pathway is the main pathway for proline synthesis in plants, while the glutamic acid pathway is the main pathway for proline synthesis in plants under stress [34]. Compared to rotary tillage, plough tillage significantly increased the grain yield and nitrogen accumulation, perhaps due to the deeper tillage depth and better soil conservation of plough tillage. Although the plough tillage cannot present the soil fragmentation as well as rotary tillage, the greater tillage depth could reduce nutrition loss by surface runoff. Therefore, in our study, the higher OAT activity observed in plough tillage might be because plough tillage increased the total nitrogen accumulation in fragrant rice, ultimately induced increased-AP production in the grain. For no-tillage, we observed that the PDH and P5CS activities were increased significantly. This result might be because the no-tillage environment caused abiotic stress to fragrant rice. Without the tilling, the soil bulk density in NT would increase and thus increase the loss of fertilizer while the higher soil bulk density would make fragrant rice root growth more difficult. Because of under those stresses, the proline in fragrant rice was primarily synthesized by the glutamic acid pathway. This idea about NT condition causing stress is supported by the lower activities of SOD, POD, and CAT and the higher content of MDA in the no-tillage treatment compared to the rotary tillage treatment in present study because MDA production in an important indicator of oxidative stress and SOD could dismutase superoxide radical whereas POD and CAT could scavenge H_2_O_2_ [21, 35]. Furthermore, the decreased grain yield and nitrogen accumulation also indicated that fragrant rice under the no-tillage condition experienced decreased growth and development compared to that under CK and PT conditions. Our results agreed with the study of Du [36], who demonstrated that no-tillage was not able to provide a better environment for grain yield in rice production. Furthermore, our results were consistent with the study of Bao [8], which documented that a degree of biotic stress could stimulate the 2-AP biosynthesis in fragrant rice.

In addition, present study appeared that PT and NT were able to promote the soil chemical and biological characteristics because they improved the organic matter content and microorganism quantity. This result might attributed to less soil fragmentation [37].

## Conclusion

Compared to CK, PT improved fragrant rice grain yield and nitrogen accumulation and induced an increase in OAT activity and led to an increase in 2-AP concentration. No-tillage also produced increased 2-AP content in grain by enhancing PDH and P5CS activities but limited yields and nitrogen accumulation in fragrant rice. Moreover, the organic matter content and soil microorganism quantity also increased due to PT and NT treatments.

## Methods

### Plant material and growing conditions

**Fig. 6:**
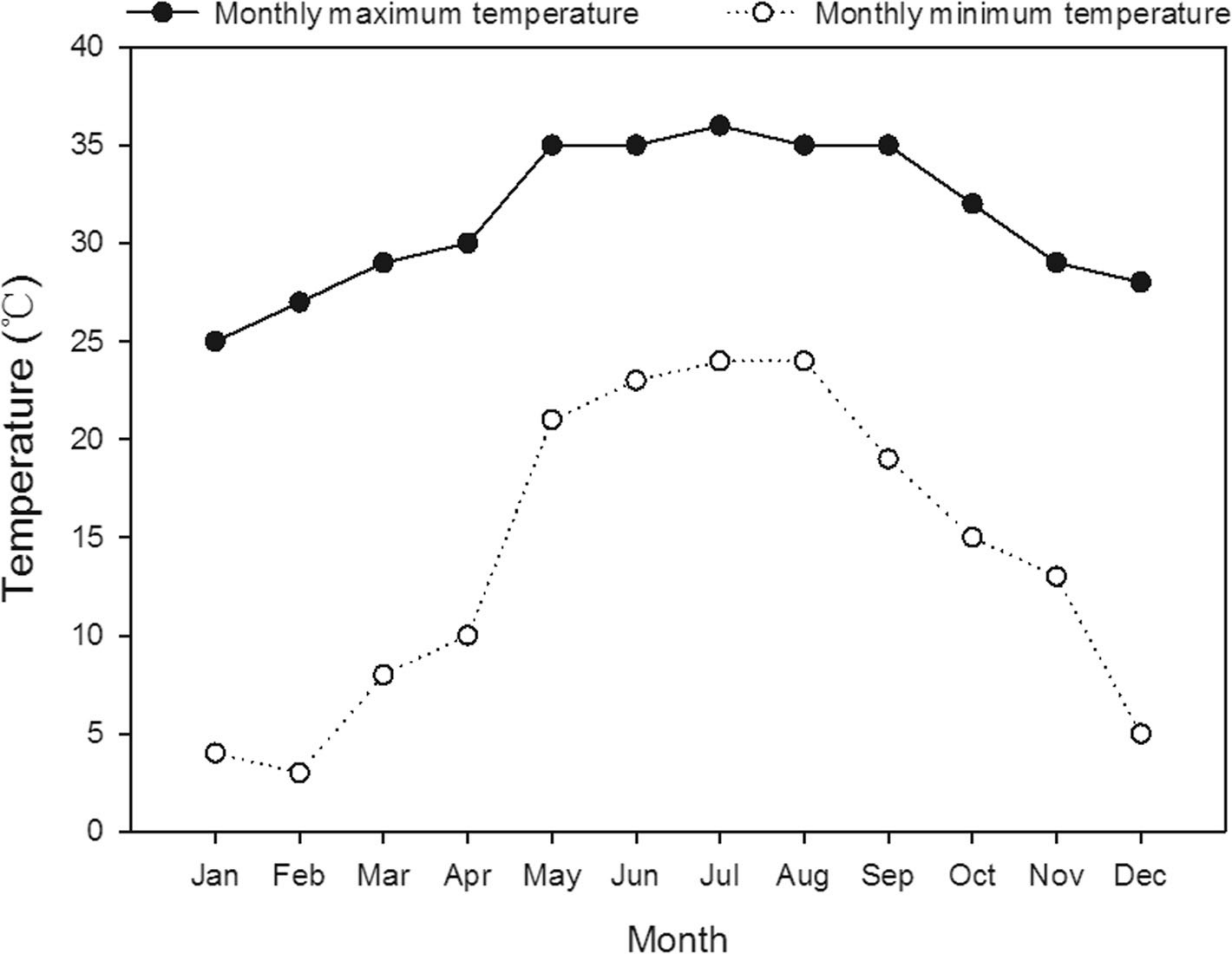
Temperature in Zengcheng in 2018

Seeds of a fragrant rice cultivar, ‘*Meixiangzhan-2*’, a well-known and widely grown fragrant rice cultivar in South China, were collected from College of Agriculture, South China Agricultural University, Guangzhou China. Before sowing, the seeds were soaked in water for 12 h at room temperature. Then, pre-germinated seeds were hill-seeded with a direct-seeded machine at a spacing of 25 × 15 cm, with 3–5 seeds planted for each hill. Two seasonal field experiments were conducted between March and November in 2018 at the Experimental Research Farm, College of Agriculture, South China Agricultural University, Zengcheng, China (23°13′ N, 113°81′ E, and 11 m from mean sea level). In both seasons, the fields were prepared with water-ponding conditions and the standing water was drained 2 days before seeding. Pre-germinated rice seeds were sown in puddled soil on March 20 and July 21 for the early and late seasons of 2018, respectively. The experimental field was under paddy cultivation for years, and the soil is sandy loam consisting of 12.27 g/kg organic matter, 0.61 g/kg total nitrogen, 53.07 mg/kg available nitrogen, 0.28 g/kg total phosphorus, 10.40 mg/kg available phosphorus, 15.63 g/kg total potassium, and 78.38 mg/kg available potassium, with a soil pH of 5.06. This region has a subtropical-monsoonal type of climate, and the temperature during the experiment is shown in Fig. 6.

### Treatments description

Two tillage methods were applied before every transplanting (with rotary tillage set as the control), as described below:

- CK (Rotary Tillage): Before seeding, the land was twice tilled with a rotary cultivator (tillage depth of 14 cm) in both seasons.
- PT (Plough Tillage): Before seeding, the land was twice tilled with a plough cultivator (tillage depth of 26 cm) in both seasons.
- NT (No-Tillage): Before seeding, there was no any tillage in the land.

The treatments were arranged in a randomized complete block design (RCBD) in triplicate each year with a net plot size of 60 m^2^. The plough cultivator was a three-furrow plough (1 L-435, China) and the rotary cultivator (1GKN-200).

### Fertilizer application and plant sampling

Special biological organic fertilizer (Dao Feng Xiang), manufactured by Guangzhou Huayuan Agricultural Ltd., China (composed of N+ P_2_O_5_ + K_2_O ≥ 74%, active living bacteria ≥20 million g − 1, and organic matter ≥10%), was applied at 900 kg ha − 1 with 60% as the basal dose and 40% at tilling. Fresh grains were collected and separated from the rice plants at 15 days after the heading stage, washed with double-distilled water, and stored at − 80 °C for physio-biochemical analysis.

### Determination of 2-AP content in grains

Fresh grains totalling approximately 0.5 g were homogenized in 5 mL of 60% ethanol and treated for 4 h in an oscillation instrument (HZS-H, China) using a frequency of 200 oscillations per minute. The ether extract was then dried over sodium sulfate, filtered (0.22 μm filter paper, Shimadzu, Japan), and then directly used to measure 2-AP concentrations with the GCMS-QP 2010 Plus (Gas Chromatography-Mass Spectrometer) method according to Huang [13], and the contents of 2-AP were expressed as μg kg^− 1^. The GCMSQP 2010 Plus working conditions were as followings: gas chromatograph equipped with a Restek Rxi-5 ms (Shimadzu, Japan) silica capillary column (30 m × 0.32 mm × 0. 25 μm). The measurements were repeated in triplicate and averaged.

### Determination of proline, pyrroline-5-carboxylic acid (P5C), and 1-pyrroline contents in grains

Grain proline concentration was estimated according to the methods of Bates [14] by using ninhydrin; the absorbance was read at 520 nm and expressed as ug g-1 fresh weight (FW) of leaves. The GABA content was measured according to the methods described by Zhao [15]. The P5C concentration was estimated following the method of Wu (Wu et al. 2009). The mixture contained 0.2 ml supernatant of enzyme extraction, 0.5 ml 10% trichloroacetic acid (TCA), and 0.2 ml of 40 mM 2-aminobenzaldehyde. After the reaction, the absorbance was read at 440 nm, and the content was expressed as μmol g^− 1^. The 1-pyrroline content in grains was estimated by the methods of Hill [16]. The amount of 1-pyrroline present in reaction mixtures initially containing 1,4-diaminobutane was determined after 30 min at room temperature. The measurements were repeated in triplicate and averaged.

### Measurement of the activity of proline dehydrogenase (PDH), △1-pyrroline-5-carboxylic acid synthetase (P5CS), and ornithine aminotransferase (OAT) in grains

PDH activity was assayed following the methods of Ncube [17]. The absorbance after reaction was read at 440 nm, and the activity was calculated using a molar extinction coefficient. The activity of P5CS was estimated according to the methods described by Zhang [18]. The reaction mixture included 50 mM Tris-HCl buffer, 20.0 mM MgCl_2_, 50 mM sodium glutamate, 10 mM ATP, 100 mM hydroxamate-HCL, and 0.5 mL of enzyme extract. OAT activity was measured according to the methods of Chen [19]. The absorbance of the supernatant fraction was read at 440 nm, and the activity was calculated by extinction coefficient 2.68 mM-1 cm-1. DAO activity was assayed by using the methods described by Su [20]. Reaction solutions (2.9 mL) contained 2.0 mL of 70 mmol L^− 1^ sodium phosphate buffer (pH 6.5), 0.5 mL of crude enzyme extracts, 0.1 mL of horseradish peroxidase (250 U mL-1), and 0.2 mL of 4-aminoantipyrine/N, N-dimethylaniline, while the activity was expressed as “U per milligram protein”. The measurements were repeated in triplicate and averaged.

### Estimation of malondialdehyde (MDA) and anti-oxidant responses

The MDA content and activities of peroxidase (POD), superoxide (SOD) and catalase (CAT) were detected according to the methods of Luo et al. [21]. After MDA reacted with thiobarbituric acid, the absorbance was read at the 532, 600 and 450 nm. The MDA content in the reaction solution was calculated as: MDA content (μmol/L) = 6.45(OD_532_ − OD_600_) − 0.56OD_450_, and finally expressed as μmol/g FW. POD (EC 1.11.1.7) activity was estimated after the reaction in the solution including enzyme extract (50 μl), 1 ml of 0.3% H_2_O_2_, 0.95 ml of 0.2% guaiacol, and 1 ml of 50 mM·l^− 1^ sodium phosphate buffer (SPB, pH 7.0). One POD unit of enzyme activity was expressed as the absorbance increase by 0.01 (U/g FW) due to guaiacol oxidation. SOD (EC 1.15.1.1) activity was measured by using nitro blue tetrazolium (NBT). In brief, 0.05 ml of an enzyme extract was added into the reaction mixture which contained 1.75 ml of SPB (pH 7.8), 0.3 ml of 130 mM methionine buffer, 0.3 ml of 750 μmol·L^− 1^ NBT buffer, 0.3 ml of 100 μmol·L^− 1^ ethylene diamine tetraacetic acid (EDTA)-2Na buffer and 0.3 ml of 20 μmol·L^− 1^ lactoflavin. After the reaction, the absorbance was recorded at 560 nm. One unit of SOD activity was equal to the volume of the extract needed to cause 50% inhibition of the color reaction. CAT (EC 1.11.1.6) activity was estimated by adding an aliquot of enzyme extract (50 μl) to the reaction solution containing 1 ml of 0.3% H_2_O_2_ and 1.95 ml of SPB and then the absorbance was read at 240 nm. One CAT unit of enzyme activity was defined as the absorbance decrease by 0.01 (U/g FW). The measurements were repeated in triplicate and averaged.

### Estimation of yield and nitrogen accumulation

At maturity stage, the rice grains were harvested from five sampling areas (2.25 m^2^) in each plot and then threshed by machine. The harvested grains were sun-dried and weighed in order to determine the grain yield. Ten representative hills of the plants were then separately sampled and divided into leaf blades, stems with sheathes, and grain. The samples were oven-dried at 80 °C (to constant weight), weighed, milled, and stored dry until analysed for total nitrogen concentration, calculated according to Pan [22].

### Estimation of soil organic matter and soil microorganism community quantity

Soil samples from 0 to 10 cm, 10–20 cm and 10–30 cm depth were taken for the determination of soil organic matter and microorganisms by the 5-point sampling method at the maturity stage in late season. The light fraction (LF) and heavy fraction (HF) of soil organic matter were separated using the method described by Camberdella and Elliott [23]. Briefly, 10 g of air-dried soil was homogenized with 30 mL NaI solution (gravity 1.8 g cm^− 3^) in a 100 ml centrifuge tube by shaking on a reciprocating shaker for 60 min at 200 rpm, after which it was centrifuged at 1000×g for 15 min. The LF, all floating material after centrifugation, was poured into a vacuum filter unit with a 0.45-μm nylon film, and the material retained by the film was washed with 0.01 M CaCl_2_ and distilled water. This process was repeated three times. The HF remaining in the centrifuge tube was washed three times with ethanol to remove excess NaI, after which it was washed twice with distilled water. Next, the LF and HF were dried at 60 °C for 48 h, and then weighed and ground to pass through a 0.15-mm sieve for the organic determinations. The organic matter in LF and HF were determined by the wet oxidation method with K_2_Cr_2_O_7_ at 170–180 °C [24]. The main soil microorganisms such as bacteria, fungi and actinomycetes were separated and counted by the dilution plate method [25].

### Statistical analyses

Experiment data were analysed using the standard analysis of variance procedure (SAS Institute, 2003). Relationships among the indexes were evaluated using correlation analyses by Statistix version 8 (Analytical software, Tallahassee, Florida, USA). The means among the treatments were compared based on the least significant difference test (LSD) at the 0.05 probability level.

## Data Availability

Not applicable.

## Abbreviations

2-AP: 2-acetyl-1-pyrroline
CAT: Catalase
MDA: Malondialdehyde
OAT: Ornithine aminotransferase
P5C: Pyrroline-5-carboxylic acid
P5CS: △1-pyrroline-5-carboxylic acid synthetase
PDH: Proline dehydrogenase
POD: Peroxidase
SOD: Superoxide dismutase
